# Risk of intracranial hemorrhage in critically ill ACLF patients: a retrospective single center cohort study

**DOI:** 10.1186/s12876-026-04875-6

**Published:** 2026-05-07

**Authors:** Miriam Dibos, Maja Wossnig, Silke Wunderlich, Roland M. Schmid, Ulrich Mayr, Tobias Lahmer, Julian Triebelhorn

**Affiliations:** 1 TUM School of Medicine and Health - Clinical Department of Internal Medicine II, TUM University Hospital, Ismaninger Str. 22, Munich, 81675 Germany; 2 TUM School of Medicine and Health - Clinical Department of Neurology, TUM University Hospital, Munich, Germany

**Keywords:** Acute-on-chronic liver failure, Intracerebral hemorrhage, Critical illness, Intensive care unit

## Abstract

**Background:**

Critically ill patients with liver cirrhosis present numerous challenges in clinical evaluation of bleeding risk. Their deficiencies in both pro- and anticoagulant factors result in a particularly fragile hemostatic system and bleeding complications. While the risk of the particular bleeding complication of intracerebral hemorrhage (ICH) is a major clinical concern, the question of whether ICH occurs more frequently in patients with acute-on-chronic liver failure (ACLF) compared to a control group and which parameters predict cerebral bleeding, remain unresolved and was the aim of this study.

**Methods:**

One hundred two critically ill ACLF patients and 166 patients in the control group were included retrospectively. Clinical parameters and occurrence of spontaneous ICH were compared to controls.

**Results:**

Cerebral computer tomography detected ICH in 15 out of 102 patients (14.7%) in the ACLF group compared to 16 out of 166 patients (9.6%) in the control group. While patients in the ACLF group exhibited prolonged prothrombin time (pTT) (median [IQR]: (57 [45–71] s vs. 42 [35–52] s, *p* < 0.001) and higher INR values (1.9 [1.5–2.4] vs. 1.2 [1.1–1.4], *p* < 0.001), significantly lower platelet count compared to control group (43 [24–64] × 10³/µL vs. 87 [39–159] × 10³/µL, *p* < 0.001) as risk factors for cerebral bleeding, statistical analysis revealed a trend towards a higher incidence among patients in the ACLF group compared to controls (OR: 1.61, chi-square-test, p-value = 0.24).

**Conclusions:**

Although statistical analysis showed a tendency to a higher incidence of ICH in the ACLF group compared to controls, ICH did not occur significantly more frequently in patients with ACLF. While no correlation was shown between the occurrence of ICH and high systolic blood pressure or dysregulated INR and pTT, low platelet counts were associated with spontaneous ICH in both groups.

**Supplementary Information:**

The online version contains supplementary material available at 10.1186/s12876-026-04875-6.

## Background

Acute-on-chronic liver failure (ACLF) is a common condition that carries a significant risk of death [1]. Especially critically ill patients with ACLF pose multiple challenges in clinical management due to their high susceptibility to various complications. One important issue in these patients is the coexistence of deficiencies in both procoagulant and anticoagulant factors, which results in a particularly fragile hemostatic system, deranged coagulation parameters and a higher bleeding tendency [2–4]. Bleeding complications such as spontaneous gastrointestinal hemorrhage, bleeding from procedures, incisions, trauma and vessel damage are well documented in cirrhosis, whereas data concerning spontaneous intracranial hemorrhage (ICH) is scarce [3].

ICH affects 44 per 100.000 cases worldwide and accounts for 10% of strokes in an overall population [5, 6]. The limited data suggests that ICH may occur more frequently in the vulnerable group of cirrhotic patients compared to non-cirrhotic controls due to the reasons mentioned above [7]. Parikh et al. reported a hazard ratio of 1.8 for ICH in cirrhotic patients versus those without cirrhosis [8]. Lagman et al. demonstrated an overall survival rate of only 14% in patients with end-stage liver disease on the liver transplant waitlist who experienced an in-hospital ICH [9]. However, data are completely lacking for the particularly vulnerable subset of cirrhotic patients requiring intensive care unit (ICU) treatment.

Primary objective of this study was to examine the incidence of ICH in critically ill patients with ACLF compared to a control group of critically ill patients without liver cirrhosis. Secondary objective was to compare the transfusion management between these two groups.

## Methods

### Data collection and study population

This retrospective study was performed in a medical intensive care unit (ICU) at Klinikum Rechts der Isar in Munich, Germany. Between January 2016 and March 2024, all patients with ACLF who received cerebral computer tomography (cCT) scan due to altered mental state, epileptic seizure or pupillary abnormalities were included. Patients with a clear trigger for ICH such as trauma were excluded from the study. Chronic liver disease was diagnosed on the basis of sonographic imaging and laboratory evidence. All of the included patients met the criteria for ACLF according to the criteria defined by the European Association for the Study of the Liver–Chronic Liver Failure Consortium [10]. Patients’ medical files were screened for age, sex, duration of mechanical ventilation, other organ failures during ICU stay (especially renal replacement therapy), ICU and hospital duration and mortality, survival at day 28 and survival at day 90. Sepsis-related organ failure assessment score (SOFA score), Child–Pugh score and ACLF score were assessed upon admission and upon cerebral imaging. Blood pressure was monitored invasively continuously by an intra-arterial catheter. The highest blood pressure within 24 h before cCT scan was recorded retrospectively and used for further analysis. Coagulation parameters (INR, pTT) and platelet count were checked daily.

This cohort was matched to a cohort of 166 non-surgical critically ill patients without liver cirrhosis and with comparable level of acute multi-organ failure who received cCT scan due to altered mental state, epileptic seizure or pupillary abnormalities.

### Statistical analysis

All statistical analyses were performed using R version 4.4.2 (R Foundation for Statistical Computing, Vienna, Austria). Distribution was assessed by Shapiro-Wilk test. Continuous variables were reported using median and interquartile ranges, due to non-normal distributed data. Categorical variables were summarized by absolute counts and percentages. t-test and the Mann–Whitney-U test were employed for the analysis of quantitative variables. Frequencies were compared by chi-square test, and fisher’s exact test if events occurred in < 5 cases. Odds ratio (OR) with 95% confidence intervals was calculated to compare risk between groups. All tests were two-sided, and a p-value below 0.05 was considered statistically significant.

## Results

### Patient characteristics

**Fig. 1:**
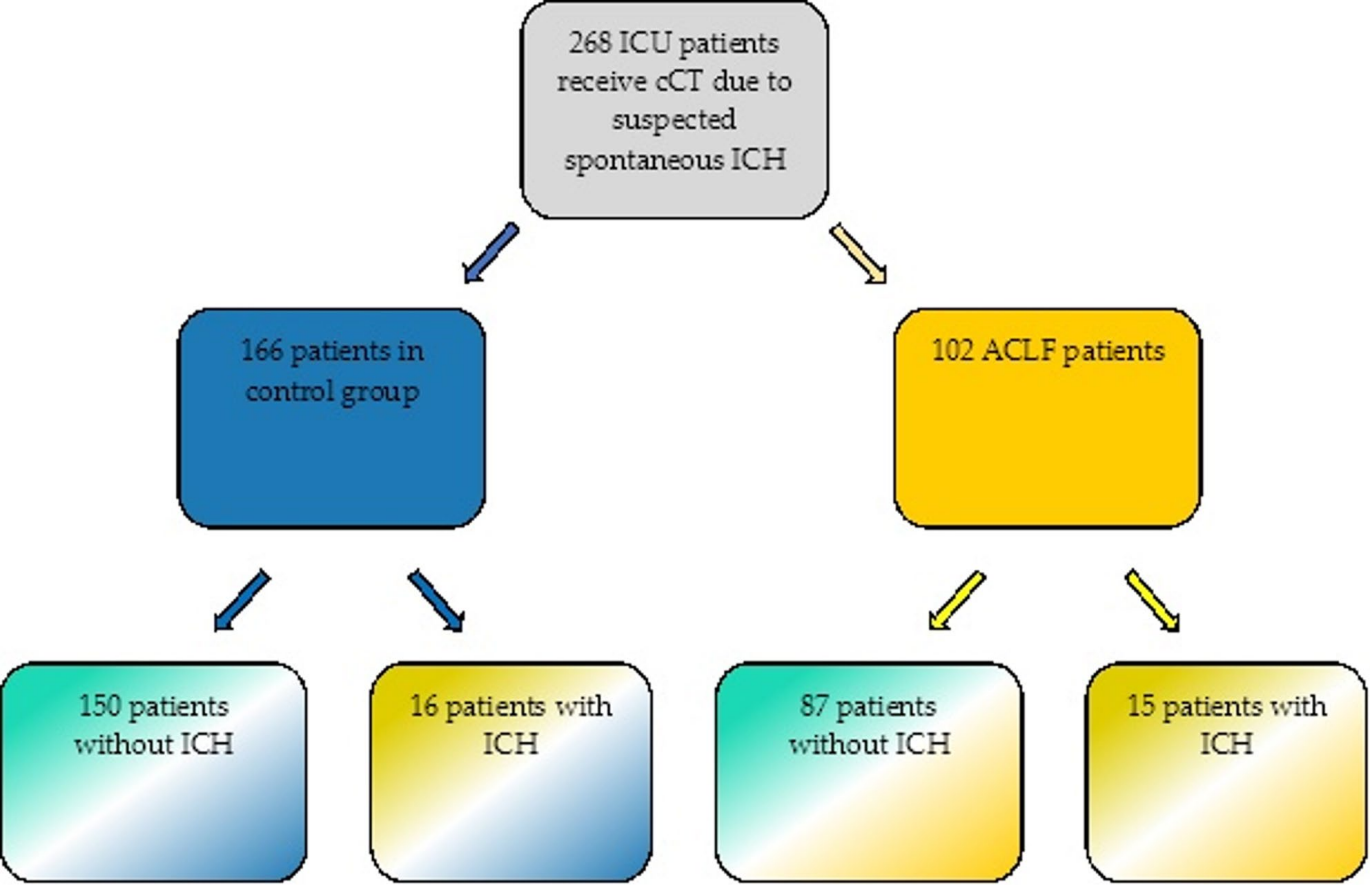
Patient selection: critically ill patients with suspected ICH who received Cerebral computer tomography (cCT). Blue color indicates control group while yellow color indicates ACLF. Green color indicates cCT imaging without pathological findings, while yellow color indicates ICH. ACLF: Acute-on-chronic liver failure, ICH: Intracranial hemorrhage

In total, 268 ICU-patients with suspected ICH who received cCT scan due to altered mental state, epileptic seizure or pupillary abnormalities were included in this study. Of these patients, 102 patients were included in the ACLF group, while 166 patients were included as controls (Fig. 1). Reasons for ICU-treatment in the control group of non-surgical ICU patients were septic shock in 42%, respiratory failure in 31%, somnolence or coma in 12%, any kind of bleeding in 6% and other reasons in 10%.

Patient characteristics are reported in Table 1. Among the patients documented, the median age in the ACLF group was 57 vs. 69 years in control group (p-value < 0.001). Etiology of liver cirrhosis was alcohol-induced in 86%, cryptogenic in 8.6%, viral in 2% and autoimmune in 3.2%. Median length of ICU stay was 21 days in the ACLF group vs. 16.5 days in the control group (p-value = 0.1), 90-day mortality in the ACLF group was 65.7% (67/102) vs. 44.6% (74/166) in the control group (p-value = 0.002).

**Table 1:** Baseline characteristics

Parameters	Control group (*n* = 166)	ACLF group (*n* = 102)	*p*-value
Age in years, median [IQR]	69 [58–77]	57 [48–67]	< 0.001
Male sex, % (*n*)	60.2% (100)	66.7% (68)	0.36
SOFA-score, median [IQR]	11 [6–14]	11.5 [8–15]	0.07
Child-Pugh-Score, median [IQR]		12 [10–13]	
ACLF-Score, median [IQR]		3 [2–3]	
Reason for cerebral imaging, % (*n*)			
- altered mental state	- 80.7% (134)	- 67.6% (69)	
- epileptic seizure	- 3% (5)	- 9.8% (10)	
- pupillary abnormalities	- 11.4% (19)	- 18.6% (19)	
- other	- 4.8% (8)	- 3.9 (4)	
Duration of mechanical ventilation, days, median [IQR]	16 [6–30]	19 [11.8–32.3]	0.05
Duration of ICU treatment, days, median [IQR]	16.5 [7-31.5]	21 [10-36.3]	0.1
In-hospital mortality %, (*n*)			
- Death day 28	- 38% (63)	- 41.2% (42)	- 0.62
- Death day 90	- 44.6% (74)	- 65.7% (67)	- 0.002
Maximum blood pressure 24 h before cCT, median [IQR]			
- systolic in mmHg	- 150 [140–175]	- 140 [130–151]	- < 0.001
- diastolic in mmHg	- 75 [70–80]	- 70 [64–80]	- 0.003
Antithrombotic medications, % (*n*)			− 0.308
- Antiplatelet therapy	- 14.5% (24)	- 7.8% (8)	
- Dual antiplatelet therapy	- 1.8% (3)	- 0% (0)	
- Therapeutic anticoagulation	- 1.2% (2)	- 0% (0)	
- Antiplatelet therapy + therapeutic anticoagulation	- 1.2% (2)	- 0% (0)	

*IQR* Interquartile range, *SOFA* Sepsis-related organ failure assessment score, *ACLF* Acute-on-chronic liver failure, *ICU* Intensive care unit

### Occurrence of ICH in dependence of group

**Fig. 2:**
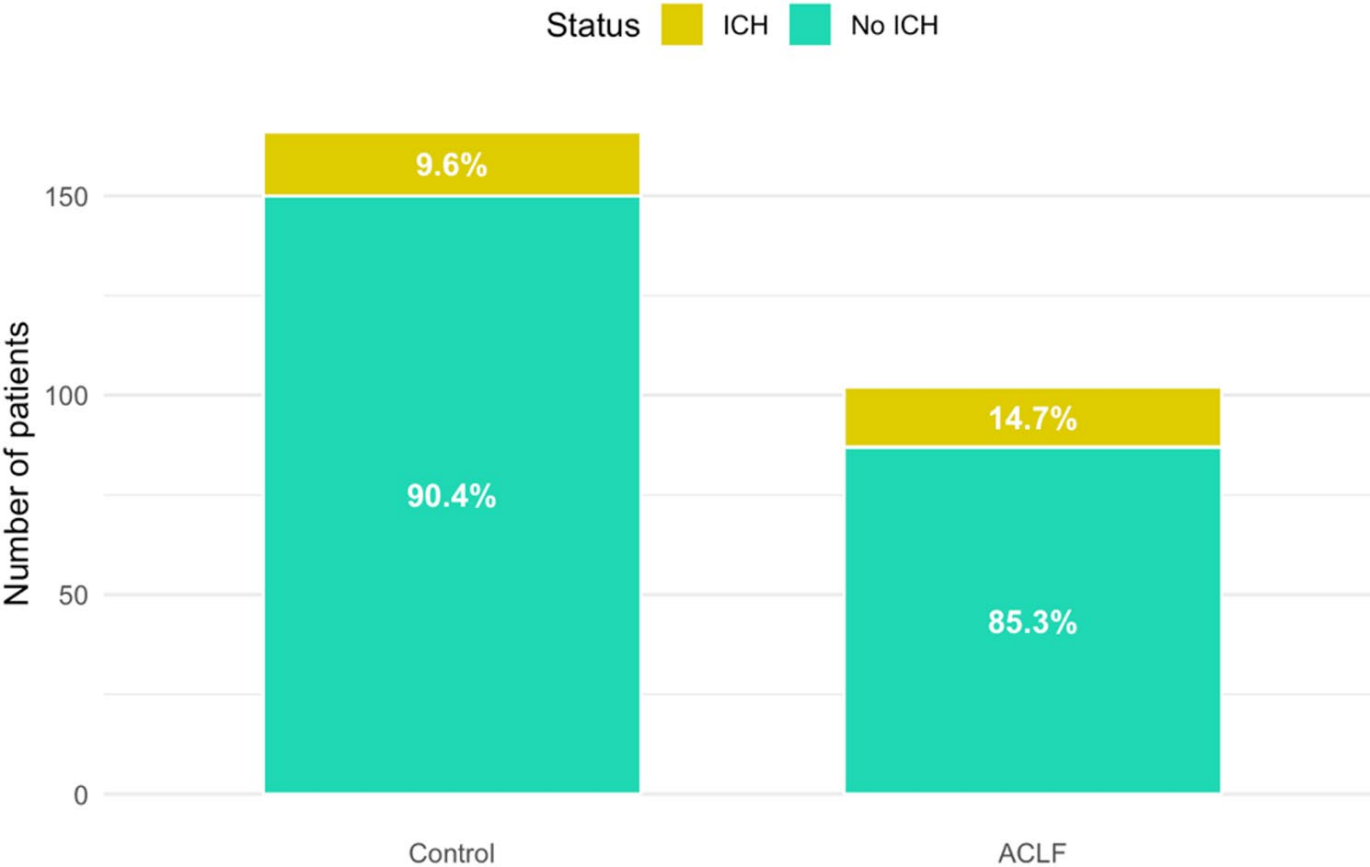
Comparison of ICH between patients with ACLF and controls. ACLF: Acute-on-chronic liver failure, ICH: Intracranial hemorrhage

15 out of 102 patients (14.7%) were diagnosed with ICH in the ALCF group (Fig. 2). Of these patients with ICH, cCT described intraparenchymal bleeding in 9 patients (60%), and subarachnoid hemorrhages or additional subarachnoid component in 10 patients (67%). Massive intraparenchymal bleeding was diagnosed by the reporting radiologist in 3 patients (20%). After neurosurgical consultation, none of the 15 patients underwent surgery.

In the control group, 16 out of 166 patients (9.6%) were diagnosed with ICH (Fig. 2). Of these 16 patients, cCT scan detected intraparenchymal bleeding in 9 patients (56.3%), subarachnoid bleeding alone or additional subarachnoid bleeding in 6 patients (37.5%) and massive intraparenchymal bleeding in 4 patients (25%). After neurosurgical consultation, 3 patients received external ventricular drainage (18.8%).

Although there was a tendency for more frequent spontaneous ICH in individuals with ACLF, statistical analysis revealed no significant difference between groups (chi-square-test, p-value = 0.24) (Table 2). The odds ratio pointed towards higher odds in patients with ACLF (OR: 1.61) compared to the control group (OR set to 1).

**Table 2:** Absolute and relative incidence of spontaneous ICH in dependence of group

	ICH	Non-ICH	*p*-value
ACLF group	15 (14.7%)	87 (85.3%)	0.24
Control group	16 (9.6%)	150 (90.4%)	

*ICH* Intracerebral hemorrhage, *ACLF* Acute-on-chronic liver failure

### Comparison of coagulation parameters between patients with ACLF and control group

**Fig. 3:**
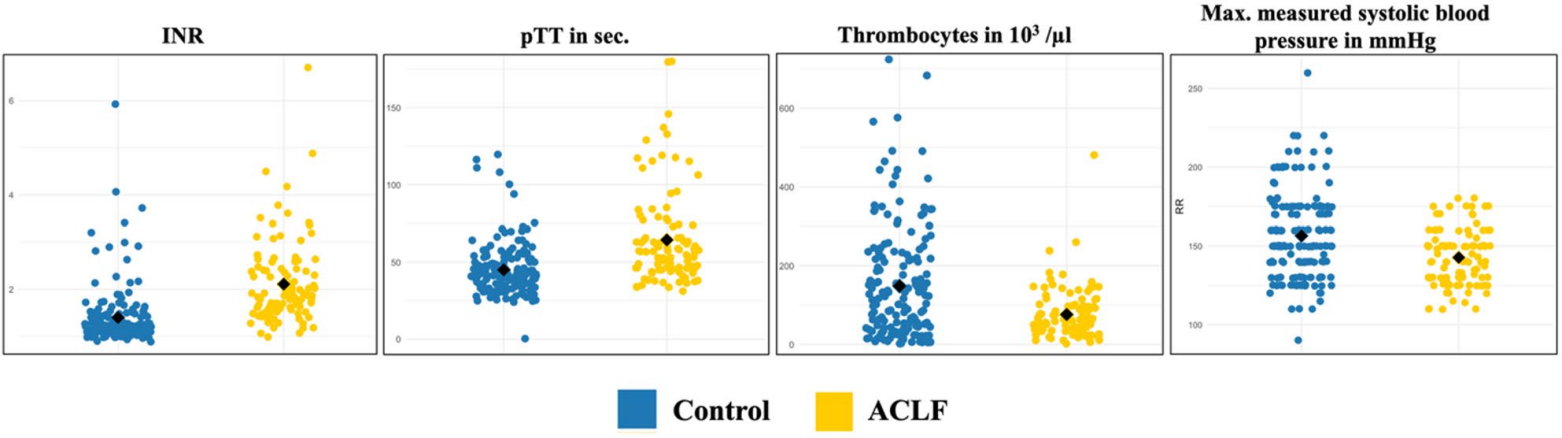
Comparison of coagulation parameters and max. systolic blood pressure between groups. ACLF: Acute-on-chronic liver failure, INR: International normalized ratio, pTT: Partial thromboplastin time

The comparison of coagulation parameters revealed significant differences between the two groups. Patients in the ACLF group exhibited prolonged prothrombin time (pTT) (57 [45–71] s vs. 42 [35–52] s, *p* < 0.001) and higher INR (1.9 [1.5–2.4] vs. 1.2 [1.1–1.4], *p* < 0.001) compared to controls. Platelet counts were also significantly lower in patients in the ACLF group (43 [24–64] × 10³/µL vs. 87 [39–159] × 10³/µL, *p* < 0.001) (Fig. 3; Table 3).

**Table 3:** Coagulation parameters prior to cCT

Parameters, median [IQR]	Control group	ACLF group	*p*-value
INR	1.2 [1.1–1.4]	1.9 [1.5–2.4]	< 0.001
pTT in s	42 [35–52]	57 [45–71]	< 0.001
Platelet count in x 10^3^/µl	87 [39–159]	43 [24–64]	< 0.001
Maximum systolic blood pressure in mmHg	150 [140–175]	140 [130–150]	< 0.001

*ACLF* Acute-on-chronic liver failure, *INR* International normalized ratio, *pTT* Partial thromboplastin time

### Comparison of max blood pressure between patients with ACLF and controls

Maximum measured systolic blood pressure within 24 h prior to cCT was significantly different between the two groups. Patients in the control group showed significantly higher maximum systolic blood pressure compared to ACLF patients (150 [140–175] mmHg vs. 140 [130–150] mmHg, p-value < 0.001) (Fig. 3; Table 3).

### Substitution of transfusion products compared between groups

**Fig. 4:**
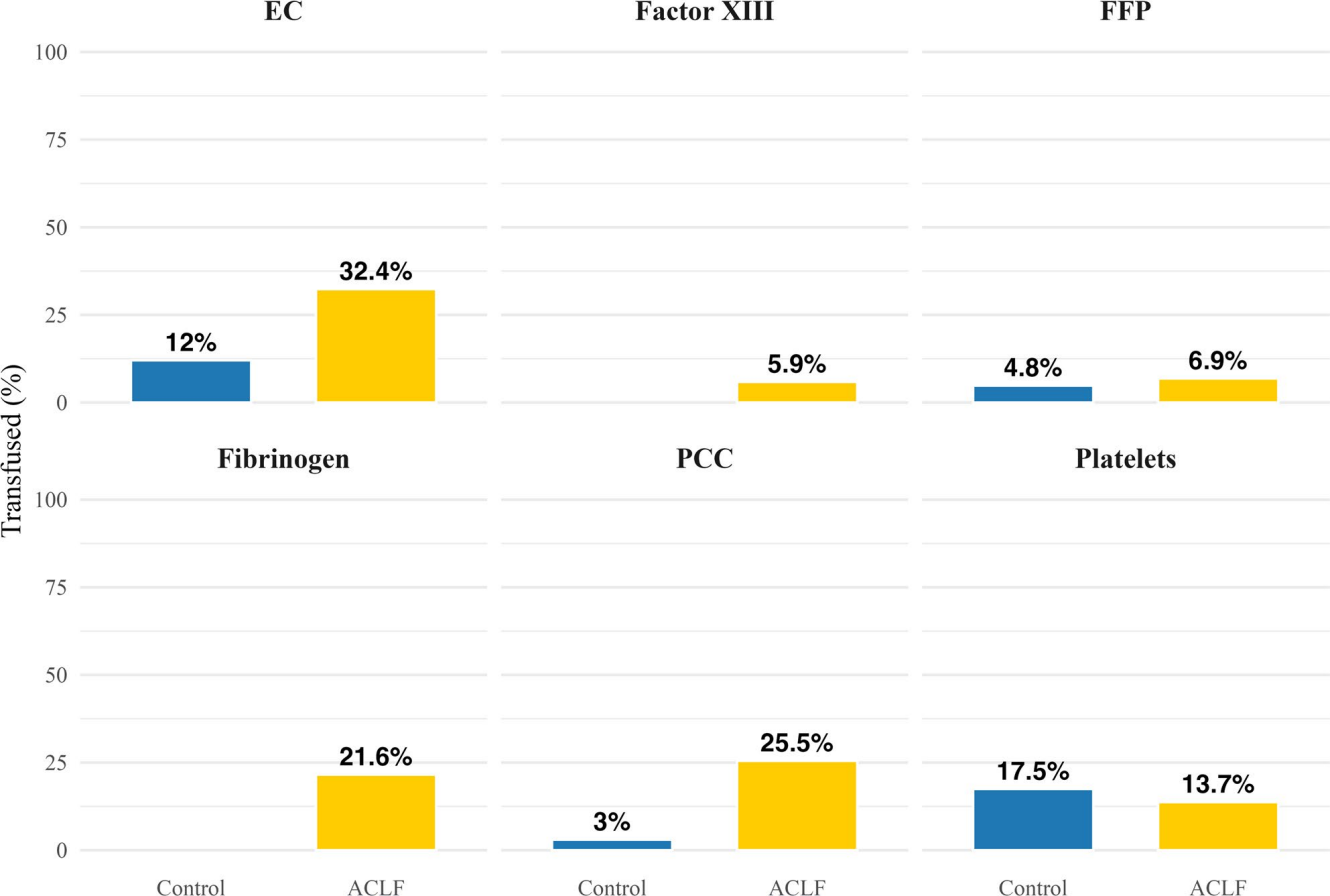
Share of patients who received blood products within 24h prior to cCT, in dependence of group. ACLF: Acute-on-chronic liver failure, EC: Erythrocyte concentrate; FFP: Fresh frozen plasma; PCC: Prothrombin complex

In the 24-hour period preceding cCT, patients with ACLF received blood products significantly more frequently than controls (erythrocyte concentrate: 32.4% vs. 12%, factor XIII: 5.9% vs. 0%, fibrinogen: 21.6% vs. 0%, prothrombin complex concentrate (PCC): 25.5% vs. 3%). Substitution of platelet concentrates (13.7% vs. 17.5%) or fresh frozen plasma (FFP) (6.9% vs. 4.8%) was not significantly different between groups (Fig. 4 and supplementary material, Table 5).

### Comparison of coagulation parameters between patients with ACLF in dependence of bleeding status

**Fig. 5:**
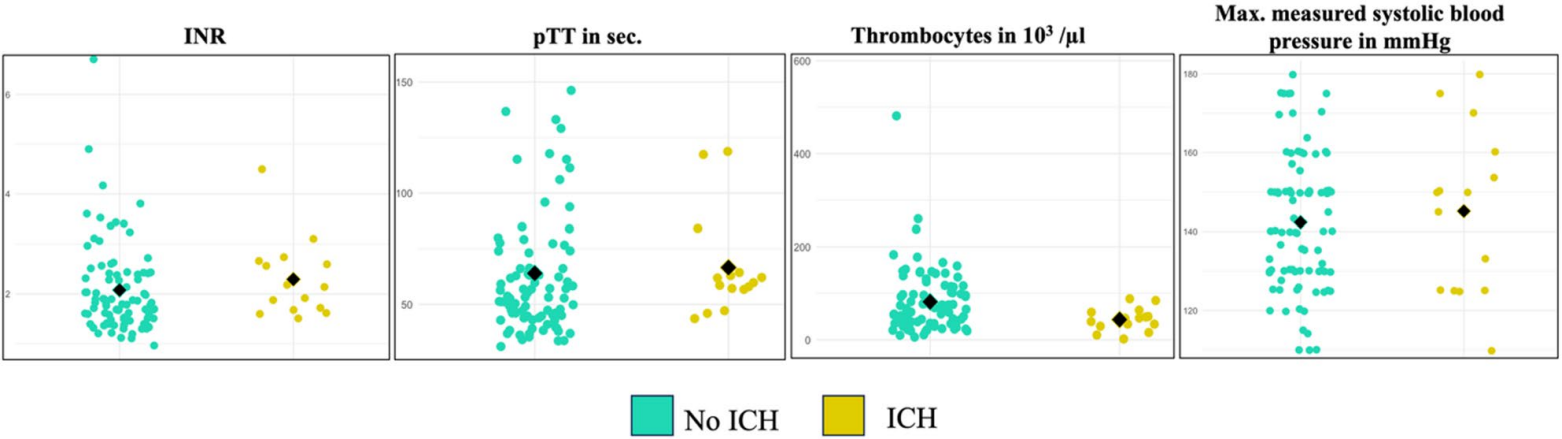
Subanalysis of coagulation parameters and maximum systolic blood pressure in ACLF-patients, in dependence on intracerebral hemorrhage (ICH) status, yellow color indicates ICH, green color indicates no ICH. ICH: intracranial hemorrhage, INR: international normalized ratio, pTT: partial thromboplastin time

Within the group of patients with ACLF, the subanalysis of coagulation parameters between patients with vs. without ICH showed that ICH was associated with a lower platelet count (46 [32–54] x 10^3^/µl vs. 66 [38–108] x 10^3^/µl, *p* < 0.001). No differences in INR or pTT were observed between patients with ACLF and with and without ICH (INR: 2.1 [1.7–2.6] vs. 1.8 [1.5–2.4], pTT: 60 [57–64] s vs. 53 [45–74] (Fig. 5).

### Comparison of maximum blood pressure between ACLF-patients with and without ICH

No significant difference was observed between maximum systolic blood pressure within 24 h prior to cCT between ACLF-patients who were diagnosed with vs. without ICH (150 [125–157] mmHg, vs. 140 [130–150] mmHg) (Table 4).

**Table 4:** Subanalysis of coagulation and maximum systolic blood pressure in ACLF-patients in dependence of ICH status

Parameters, median [IQR]	ICH	Non-ICH	*p*-value
INR	2.1 [1.7–2.6]	1.8 [1.5–2.4]	0.35
pTT in s	60 [57–64]	53 [45–74]	0.71
Platelet count in x 10^3^/µl	46 [32–54]	66 [38–108]	< 0.001
Maximum systolic blood pressure in mmHg	150 [125–157]	140 [130–150]	0.63

*ICH* Intracerebral hemorrhage, *INR* International normalized ratio, *pTT* Partial thromboplastin time

### Substitution of blood products in dependence of spontaneous intracranial hemorrhage

**Fig. 6:**
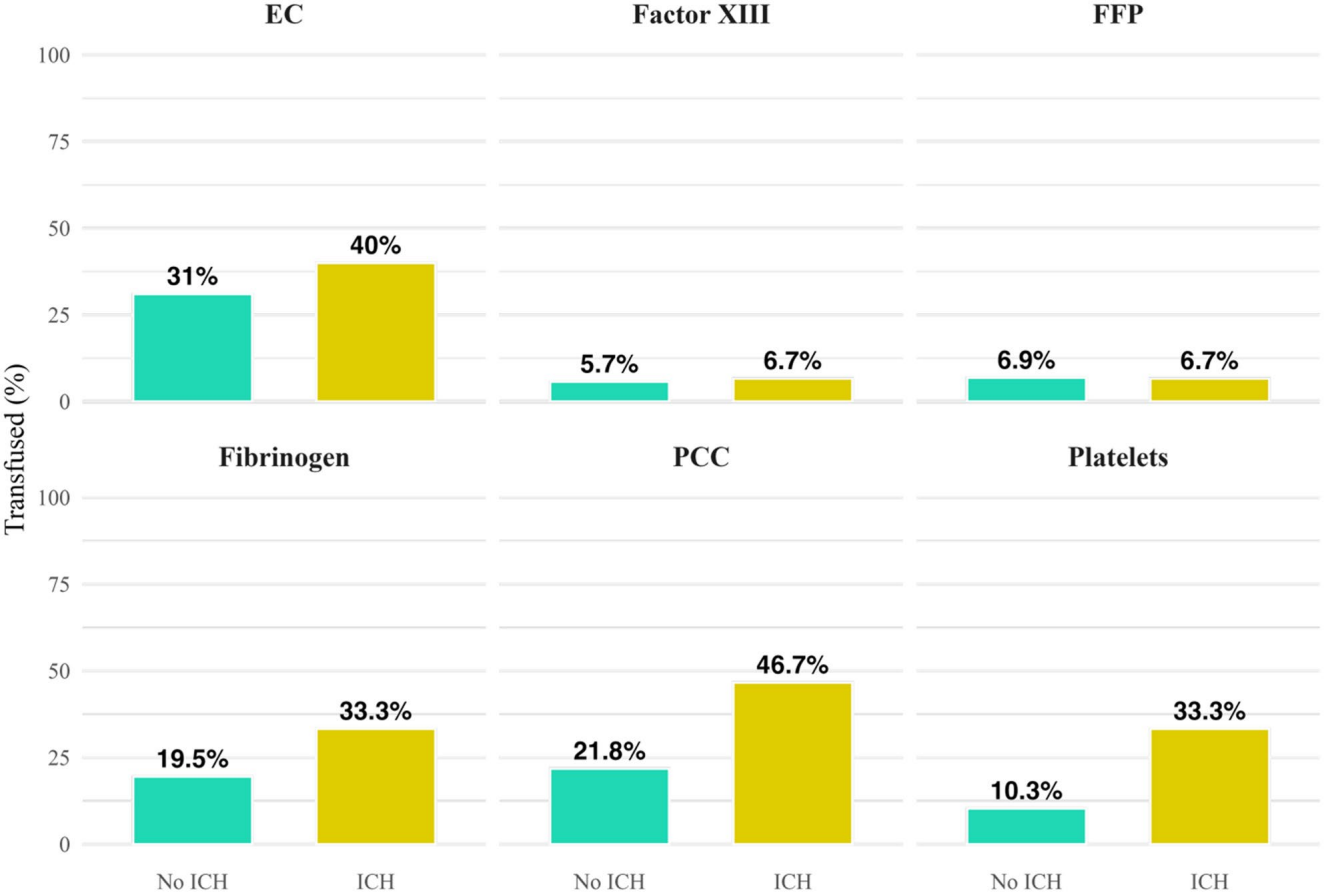
Share of ACLF-patients who received blood products 24h prior to cCT, in dependence of ICH status. ACLF: Acute-on-chronic liver failure, EC: Erythrocyte concentrate; FFP: Fresh frozen plasma; ICH: Intracranial hemorrhage, PCC: Prothrombin complex

ACLF patients with ICH received significantly more platelet concentrates compared to ACLF patients without ICH within 24 h prior to cCT (ICH: 33.3% vs. non-ICH 10.3%, *p* = 0.047, Fig. 6, supplementary materials, Table 6). No significant difference was observed between patients with and without ICH in regards to transfusion of erythrocyte concentrates, factor XIII, fresh frozen plasma, fibrinogen or PPC (Fig. 6 and supplementary materials, Table 6).

## Discussion

The main finding of this retrospective analysis was a tendency toward a higher incidence of ICH in the ACLF group (14.7%) compared with the control group (9.6%), although the difference was not statistically significant. Despite significant coagulation disorder (INR, pTT, platelet count) in the ACLF group, ICH did not occur significantly more frequently which may be attributable to the small sample size.

Leading risk factors for ischemic and hemorrhagic strokes are hypertension and high systolic blood pressure in the acute situation, followed by smoking, alcohol consumption and abuse as well as oral anticoagulation and coagulation disorder in an overall population [6, 11]. Concerning high systolic blood pressure, Holt Jahr et al. compared systolic blood pressures in different types of stroke and showed that patients with ICH had the highest systolic blood pressure before ICH (173 ± 34 mmHg) compared to other types of stroke, which was higher compared to the systolic blood pressure in both our groups (140 [130–150] mmHg in ACLF group vs. 150 [140–175] mmHg in control group) [12].

Another main risk factor for ICH is coagulopathy, as mentioned in the currently available guidelines of ICH [6]. However, these guidelines do not address patients with coagulopathy secondary to impaired hepatic synthesis, but only refer to ICH secondary to coagulopathy due to oral anticoagulation or antiplatelet therapy [5, 6, 13, 14]. In these cases, they suggest vitamin K (in cases of vitamin-k-antagonist) and the substitution of PCC [6]. Moreover, transfusion of platelet concentrates in case of spontaneous ICH associated with antiplatelet therapy is not recommended [6]. In our study, patients in ACLF group presented with significantly lower coagulation parameters (such as INR, pTT and platelet count) compared to the control group as is known for ACLF in the literature [4, 15]. Additionally, when comparing the amount of blood products substituted within 24 h before CT scan, patients with ACLF required significantly more blood products except for platelet concentrates. This even further emphasizes the extent of coagulation disorder, as current clinical recommendations for patients with liver cirrhosis generally advise against routinely correcting coagulopathy and thrombocytopenia [16]. Instead, these guidelines recommend sparing use of blood products as these blood products increase portal pressure and carry a risk of transfusion-associated acute lung injury and circulatory overload [16].

Within the group of patients with ACLF, ICH was associated with a lower platelet count. Also, ACLF-patients with diagnosed ICH received platelet concentrates significantly more often within 24 h prior to cCT. Thrombocytopenia has already been described by others as being associated with ICH: Mrochen et al. performed a multicenter retrospective cohort study investigating 2,252 patients with ICH with accompanying thrombocytopenia in 13.1% and found worse outcomes for patients with thrombocytopenia [17]. In fact, patients with thrombocytopenia < 100 G/l were excluded in this study, while in both groups included in our study, median platelet count in patients with ACLF was 43 [24–64] x 10^3^/µl versus 87 [39–159] x 10^3^/µl in control group [17]. Rather than serving as a prognostic marker, low platelet count might be associated with an increased bleeding risk in this vulnerable cohort as well as a marker of spontaneous ICH-risk in critically ill patients with ACLF. However, the amount of platelet and factor replacement often reflects clinician concern rather than genuine modifiers of bleeding risk.

This study has multiple limitations. As a retrospective single-center study it is susceptible to selection-bias. Furthermore, the small size of the two compared groups limits the meaningfulness and generalizability of the results and a lack of statistical power (power (1-ß error probability) = 0.25). 508 patients would be required in the ACLF group and 833 in the control group to achieve adequate statistical power. Moreover, thrombocytopenia occurred in the ACLF as well as the control group, as our control group consisted of non-surgical critically ill, often septic patients or patients with hematological underlying diseases. However, this is a realistic clinical scenario. Additional future studies with larger sample sizes and prospective designs could help clarify whether platelet transfusion helps reduce the risk of ICH in patients with low platelet count. Furthermore, coagulation management should be guided by viscoelastic testing (e.g. ROTEM) in addition to conventional laboratory parameters including absolute platelet count and measurement of adenosine diphosphate (ADP).

## Conclusion

In summary, while patients in the ACLF group exhibited coagulopathy, statistical analysis only revealed a trend towards a higher incidence among patients in the ACLF group compared to controls (OR: 1.61, chi-square-test, p-value = 0.24). Therefore, when critically ill patients with or without liver cirrhosis develop neurological symptoms in addition to coagulopathy, cCT should be performed immediately. No correlation was seen between the occurrence of ICH and either high systolic blood pressure, or the dysregulation of INR and pTT, emphasizing the necessity for other approaches to assess coagulopathy and hemorrhagic risk in cirrhotic patients. Low platelet count and transfusion of platelet concentrates within 24 h prior to cCT was associated with spontaneous ICH in the ACLF group. Further studies are needed to develop guidelines for the management of thrombopenia in this particularly vulnerable demographic.

## Supplementary Information

Supplementary Material 1.

## Data Availability

The datasets used and/or analysed during the current study are available from the corresponding author on reasonable request.

## Abbreviations

ACLF: Acute on chronic liver failure
cCT: Cerebral computer tomography
EC: Erythrocyte concentrate
FFP: Fresh frozen plasma
ICH: Intracerebral hemorrhage
INR: International normalized ratio
ICU: Intensive care unit
OR: Odds ratio
PCC: Prothrombin complex
pTT: Prothrombin time
ROTEM: Rotational thromboelastometry
SOFA: Sepsis-related organ failure assessment score
