# Supplementary material for: Risk of intracranial hemorrhage in critically ill ACLF patients: a retrospective single center cohort study
Source: BMC Gastroenterol. 2026 May 7;26:281. doi: 10.1186/s12876-026-04875-6 (PMC13154455; doi:10.1186/s12876-026-04875-6)
Supplement: Supplementary file 1 — Supplementary Material 1. [file 12876_2026_4875_MOESM1_ESM.docx]

**Supplementary materials**

Table 5: comparison of substitution between ACLF group and control

| Substitution | Substituted in % of patients in ACLF group | Substituted in % control | Test | p-value |
| --- | --- | --- | --- | --- |
| Erythrocyte concentrates | 32.4 | 12 | Chi-square | <0.001 |
| Factor XIII | 5.9 | 0 | Fisher exact | 0.003 |
| Fresh frozen plasma | 6.9 | 4.8 | Fisher exact | 0.665 |
| Fibrinogen | 21.6 | 0 | Fisher exact | <0.001 |
| PPC | 25.5 | 3 | Fisher exact | <0.001 |

Table 6: Share of ACLF-patients who received blood products, 24h prior to cCT, in dependence of ICH status

| Substitution | Substituted in % of patients with ICH | Substituted in % of patients without ICH | Test | p-value |
| --- | --- | --- | --- | --- |
| Erythrocyte concentrates | 40 | 31 | Chi-square | 0.7 |
| Factor XIII | 6.7 | 5.7 | Fisher exact | 1 |
| Fresh frozen plasma | 6.7 | 6.9 | Chi-square | 1 |
| Fibrinogen | 33.3 | 19.5 | Chi-square | 0.39 |
| PPC | 46.7 | 21.8 | Chi-square | 0.09 |
| Platelet concentrates | 33.3 | 10.3 | Chi-square | 0.047 |
